# Dynamics on the field: a focused study on the culture and context of pediatric pain management at four Ghanaian hospitals

**DOI:** 10.1186/s12887-020-02399-w

**Published:** 2020-11-20

**Authors:** Abigail Kusi Amponsah, Evans Oduro, Victoria Bam, Joana Kyei-Dompim, Collins Kwadwo Ahoto, Anna Axelin

**Affiliations:** 1 Department of Nursing Sciences, Faculty of Medicine, University of Turku, Turku, Finland; 2 Department of Nursing, Faculty of Allied Health Sciences, Kwame Nkrumah University of Science and Technology, Kumasi, Ghana

## Abstract

**Background:**

As part of efforts to develop and implement a short course educational program on pediatric pain management, the current study sought to understand the culture and contextual factors that influence children’s pain management in order to improve the practice in pediatric care settings.

**Methods:**

Guided by Bourdieu’s theory of practice, a focused ethnographic study was conducted from October, 2018 to February, 2019. The study was contextualized at four Ghanaian hospitals among purposefully sampled nurses, physicians, hospitalized children and their families. During the 20-week study period, three ethnographers spent 144 h conducting participant-observation sessions. Formal and informal interviews were held with participants in addition to review of hospital records.

**Results:**

Analysis of the field data resulted in four themes. “Children’s pain expression and response of caregivers” described the disposition *(habitus)* of both children and caregivers to act in particular ways due to children’s incomplete health status *(bodily capital)* which caused them pain and also resulted in discomforting procedures. “Pharmacological pain management practices and attitudes” elucidated the use of analgesics as the mainstay disposition *(habitus)* in children’s pain management due to high level of respect *(symbolic capital)* given to such interventions on the pediatric units *(field)*. “Managing pain without drugs” illustrated healthcare providers and family caregivers’ disposition *(habitus)* of using diverse nonpharmacological methods in managing children’s pain*.* “Communication and interaction between pain actors” depicted how children’s access to care givers (*social capital)* can serve as a powerful tool in influencing pediatric pain assessment and management disposition *(habitus)* on the pediatric units *(field)*.

**Conclusions:**

The *habitus* of pediatric pain actors toward pain assessment and management practices are influenced by various forms of *capital (social, cultural, symbolic, bodily and economic)* operating at different levels on the pediatric care *field.* Quality improvement programs that seek to enhance pediatric pain management should use the insights obtained in this study to guide the development, implementation and evaluation stages.

**Supplementary Information:**

**Supplementary information** accompanies this paper at 10.1186/s12887-020-02399-w.

## Background

Many hospitalized children continue to suffer needless pain [1–3] due to a myriad of factors such as insufficient resources, caregivers’ limited competencies, cultural issues, context of pain management among others [4, 5]. The effects of unrelieved pain among children cannot be overemphasized. Inadequately treated pain can lead to both short-term and long-term biopsychosocial consequences on affected children. These include: activation of the stress response [6], impaired functional ability [7], delayed recovery, prolonged hospitalization, increased cost of healthcare [8], absence from school [9], post-traumatic stress [10], isolation [11], chronic pain and negative impact on children’s quality of life [12]. Unrelieved children’s pain also affects the social, psychological and financial aspects of the family and society [13].

Considering the negative consequences of unrelieved pain, it is not surprising that suboptimal pain management has been considered an international health tragedy [14] and the freedom from pain regarded as a basic human right [15]. Furthermore, the International Association for the Study of Pain [15] declared the right of all people to have access to nondiscriminatory pain management, the right to pain acknowledgment and information on its assessment and management, as well as the right to have access to appropriately trained healthcare professionals. In order to prevent the numerous associated effects of undertreated pain, the American Academy of Pediatrics also advocates for the expansion of pediatric pain assessment and management knowledge among healthcare providers [16].

As part of efforts to improve pediatric pain management, several guidelines have been developed to guide the assessment and management of children’s pain [17–19]. In spite of these best practice guidelines, pain management largely remains under-prioritized in children [4, 20]. The under-prioritization of children’s pain management may be the result of failure to translate best practice guidelines into practice, demonstrating the importance of context in implementation science [21]. Earlier studies have demonstrated the important value of cultural context in shaping evidence-based practices by caregivers in the assessment and management of pain in pediatric care settings [22, 23].

Culture refers to the behaviour, attitudes, values, systems of meaning and skills shared by a group of people [24]. It guides beliefs and attitudes pertaining to meaning of illness, healthcare seeking behaviours, degree of receptivity to healthcare interventions, and healthcare practices [25, 26]. The sociocultural context is critical in enhancing our understanding of pediatric pain management, especially in low-middle income countries where culture has been identified as a barrier to optimal children’s pain management [27, 28]. Although efforts have been channeled toward addressing the knowledge, attitudes, self-reported practices, impact of pain care [29], experiences and perceptions of stakeholders involved in children’ pain management [30]; there is limited evidence on the context and cultural factors that underpin the assessment and management of pediatric pain in clinical practice.

Healthcare in the developing country named Ghana is provided and regulated by the Ministry of Health (MoH) and its affiliated bodies and agencies such as the Ghana Health Service and the Nursing and Midwifery Council [31]. Majority of the citizens are enrolled unto a national health insurance scheme that enables them to access a health services through yearly payments, but this system has not been able to completely eliminate out-of-pocket payments, thereby making it difficult for patients, who are mostly poor to afford some services such as pain medication [32]. In addition to insufficient nurse-patient and doctor-patient ratios of 1:542 and 1:8481 respectively [31], health resources such as equipment and tools are very limited and are unevenly distributed, doubling the plight of patients in district and rural centres. However, healthcare in Ghana incorporates a strong familial presence and a sense of cordiality, empathy and understanding between patients, family caregivers and their healthcare personnel [33]. Unfortunately, majority of nursing staff who cater for hospitalized children are not paediatric nursing specialists [34] and this contributes to insufficient pediatric pain management competencies among children’s nurses in Ghana [35, 36].

Earlier studies have enhanced our understanding on the influence of context in shaping the complexity involved in pain management in clinical settings [23, 37]. It appears from the reviewed literature that, the culture and context of pediatric pain management has not been explored from Sub-Saharan African perspective. The effectiveness of healthcare practice is also highly dependent on the resources available to the practitioner as well as the environment and culture that forms the framework for interaction with the patient [38]. As part of efforts to develop and implement a short course educational program on pediatric pain management, the current study sought to understand the culture and contextual factors that influence children’s pain management in order to improve the practice in these settings.

## Methods

### Study design

Guided by Bourdieu’s theory of practice [39], a focused ethnographic study was conducted over the course of 5 months from October, 2018 to February, 2019. This approach was chosen as the researchers intended to understand the processes involved in the assessment and management of children’s pain within the pediatric care settings of four Ghanaian hospitals through the use of three foundational concepts (field, capital and habitus). According to this theory, *field* refers to the social space and structures within which individuals practice. *Capital* signifies resources or power over a field and the individuals operating within it; this power may take the form of *social* (peers, networks), *cultural* (education, socio-demographic), *economic* (salary, finances), bodily (health status) or *symbolic* (reputation, respect, status). *Habitus* represents dispositions or inclinations which cause individuals to behave in particular ways over time and is demonstrated through an ongoing and emerging relationships between the individual (agency) and the collective (structure); it is underpinned by personal experiences, backgrounds, professions and circumstances.

The overall goal of this focused ethnographic study was to gain an understanding of both the emic and etic perspectives. Emic perspective describes how members or insiders of a particular group perceive and understand their world whereas the etic perspective characterizes an outsiders’ understanding of an observed culture [40]. The present study formed part of a larger study that sought to explore the educational needs on pain management in children.

### Setting

The study was contextualized at four hospitals in the Ashanti region of Ghana. For the purposes of ensuring anonymity and confidentiality, the four hospitals have been labelled as A, B, C and D. These hospitals were purposively chosen as they all had specially designed in-patient children’s care settings and were located in diverse geographical locations (urban, peri-urban, rural). Hospital A was a specialist private children’s hospital with a bed capacity of 20. Hospital B was also a specialist children’s hospital owned by the government of Ghana; it had 26 beds in the facility. Hospital C was a quasi-governmental hospital with a 22-bed capacity pediatric unit. Hospital D was a mission-based hospital with 19 beds in the pediatric unit. All four hospitals admit children under 13 years and oversee to those with medical or minor surgical conditions.

The pediatric care settings were colourfully painted and had drawings of flowers, fruits, cartoons, rainbows, balloons among others. The units were divided into various sections to cater for children based on their age or disease condition. There were specially designated nurses’ station where newly admitted children and their families were received and examined. The units also had an emergency or resuscitation area where they triaged and cared for children in need of such services. The in-patient beds had a side locker for keeping patients’ belongings and a bedside chair for the parent or guardian. As part of the hospital protocols, hospitalized children are housed with at least one parent or guardian at all times in the pediatric units. However, the hospital had limited accommodation facilities for these parents or guardians. All the pediatric care settings had notice boards on which the ward protocols and duty rosters were displayed.

During the period of the study, the weekly admission rates within the children’s unit ranged from six to 15. The pediatric care settings are staffed by 10–20 nurses and three to six physicians who work on a shift basis. The number of nurses per shift ranges from two to six and that of physicians varies from one to two. Nursing work within the children’s unit was daily operationalized on a three-tier shift system: morning, afternoon or night duties. This system ensured the provision of a 24-h continuous nursing care to hospitalized children and their families. On a daily basis, physicians visited the wards individually or as a team to review the conditions of the admitted children. Physicians are also consulted outside their working hours as and when deemed necessary.

### Participants

Participants for the current study comprised of nurses, physicians, hospitalized children and their families. Nurses and physicians were purposefully sampled if they were working in the pediatric units of the included hospitals. Hospitalized children were selected if they had pain complains as one of the symptoms of their present medical condition or were undergoing an invasive or skin-breaking procedure. Families of such children were also purposively sampled to participate in the study.

### Data collection procedures

Data collection for the study began following administrative and ethical approvals from the respective hospitals and ethics committee. The researchers approached the nurse managers and the nurse-in-charges of the children’s units and briefed them about the purpose and procedures involved in the study. The nurse in-charges then introduced the researchers to eligible nurses, physicians, hospitalized children and their families. The researchers then briefed the eligible participants on the scope of the research and gave them the opportunity to ask questions. Answers and clarifications were given to participants before the study began and during the entire research period. Reflexivity was ensured by keeping a detailed journal during data collection and analysis, where personal reflections and perceptions were written so that both the emic and ethic experiences did not bias the participants’ accounts. Prior to each fieldwork, the ethnographers (AKA, JKD & CKA) documented their mood and expectations before entering the unit to serve as a check when identifying the research themes at a latter period. Field data for the current study comprised of observations, interviews (formal and informal) and review of documents (audit).

### Observations

Observational data collected within the first three sessions (morning, afternoon, evening) in the children’s unit of each hospital were not used in analysis as researchers (AKA, JKD & CKA) wanted some familiarization with the participants in order to reduce changes in participant’s behaviour as a result of being observed [41]. At the beginning of each day’s fieldwork, the researchers took part in friendly conversations and other non-pain-related activities with the nurses, physicians, children and their families. The researchers actively kept a moderate level of participation by balancing participation with observation [42]. This approach enhanced co-operation and a positive working relationship with the participants throughout the data collection period.

Two of the ethnographers were female nurses and the third was a male nurse, all of whom did not work in any of the included hospitals. They entered the children’s units, wearing their nursing uniforms and blended in as natural participants [43]. The ethnographers participated in daily nursing activities as shadow nurses whilst keenly observing and documenting activities related to children’s pain assessment and management. The ethnographers were present at each of the hospitals for 2 to 3 days per week and, on average for 4 h per each observation. During the 20-week study period, the ethnographers spent 144 h conducting participant-observation sessions with 36 h spent in each hospital.

Observations were guided by a checklist to keep the researchers’ focus on children’s pain assessment and management. Specifically, observations were focused on the ward environment, number of participants present during each observation (nurses, physicians, number of children on admission, families of hospitalized children), pain assessment, pharmacological and nonpharmacological pain management activities, and documentation of pain assessment and management interventions, as well as the feelings of all pain actors present in the unit. In addition, data on the availability of pain tool, its location and interactions among the participants (nurses, physicians, children and families) were also observed.

### Formal interviews

Twenty-eight (28) nurses and 12 physicians working in the children’s unit of the included hospitals were purposively chosen to participate in the formal interviews. Consideration was given to age, gender, working years in the health profession and in the children’s unit. Formal interviews were held each with hospitalized children who were above 5 years (20) and their corresponding family member who was resident with them in the hospital (20). Apart from the child’s ability to communicate effectively, the selection process considered the gender, age, medical condition or procedure performed. Efforts were also made to include diverse categories of family care-givers such as mothers, fathers, grandmothers, aunties, uncles among others. The children, family caregivers, nurses and physicians from the four different hospitals were purposefully selected to achieve maximum variation to enhance our understanding on the socio-cultural context of pediatric pain management in Ghana.

Individual or group interviews were conducted on scheduled dates with the participants in English or Asante Twi languages (a popular indigenous dialect spoken in Ghana) language. The interviews were recorded with participants’ permission and lasted from 10 to 40 min per each session. Individual interviews with children lasted between 10 to 20 min, those with adult interviews lasted from 10 to 30 min; group interviews of families and healthcare providers lasted between 20 to 40 min.

With the aid of a semi-structured guide (Additional file 1: Appendix I, II and III), four authors (AKA, JKD, EO and CKA) facilitated the interview sessions at private, quiet rooms within the hospital premises. At least two out of the four authors were present during each interview to allow for notes taking and the smooth running of the sessions. Notwithstanding, the first author (AKA) was present at all interviews either as a facilitator or notes taker during the sessions. All interviews were completed in one hospital before moving to the next.

The nurses, physicians and family care givers responded to questions which included: their views on the prevalence of children’s pain, methods they used in assessing and managing children’s pain, their communication and preparedness towards their role in children’s pain assessment and management. The hospitalized children were also asked similarly about the extent of pain they experience as a result of their medical conditions or procedures performed on them. They were also asked about the role the other participants (nurses, physicians, family care givers) played in the assessment and management of their pain and their wishes regarding pain care. Recorded interviews were transcribed verbatim by two of the researchers (EO and CKA) after which they were analyzed by all the researchers involved in the study before the next data collection session. The interviews that were conducted using the Asante Twi language were translated into English and back translated into the original language with the assistance of a language translator.

### Informal interviews

Informal interviews were casual conversations, in which the ethnographers clarified observations with participants. These were noted in shorthand by the researchers in their field notes and later transcribed in full for analysis. Forty (40) nurses, 12 physicians, and 72 children-family dyads were informally interviewed over the study period.

### Document audits

Over the course of 5 months, a total of 108 patient folders were reviewed for pain assessment and management reports. Patient folders of those children who had pain complains during hospitalization and had been on admission in the unit for at least 24 h were selected. Thirty-six (36) nursing reports and physician notes on hospitalized children’s conditions were thoroughly examined over the same time period. Documents displayed on the notice boards of the wards and the pediatric care settings were also reviewed.

### Data analysis

**Fig. 1:**
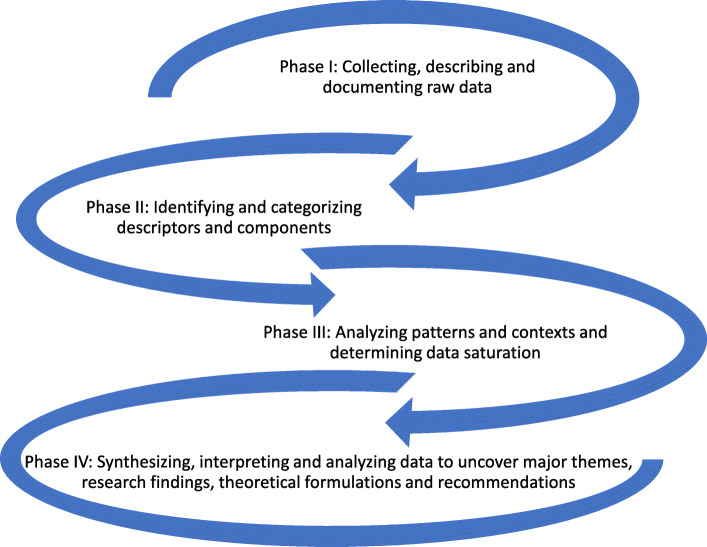
Four phases of Leininger’s data analysis

Using Bourdieu’s theory of practice [39] as the theoretical lens, an iterative process of data collection and inductive analysis followed the data analysis method developed by Leininger [44] (refer to Fig. 1). Recorded field notes, transcribed interviews and document review reports were transported into NVivo 12 Plus software for data management. Identification of themes was carried out by reading through field notes, transcripts and document review reports of each study site multiple times. Coding was conducted by the ethnographers and verified with the assistance of two experienced qualitative researchers. Concepts and themes were continuously clarified, revised and updated during data collection and analysis period. Documented personal reflections were consulted in the data analysis process to ensure that the researchers’ experiences did not bias participants’ accounts. Data saturation was achieved within and across study sites as no new information or themes emerged from the field data. Information gathered during the fieldwork were merged during thematic analysis to create a holistic sense of the culture and context of pain assessment and management in the children’s units of hospitals in Ghana.

### Trustworthiness

The trustworthiness of this focused ethnographic study was ensured by adhering to Guba and Lincoln’s [45] principles of credibility, conformability, dependability and transferability. Credibility and conformability were achieved through triangulation and member-checking. Triangulation was enhanced using multiple study settings (four hospitals with different ownership and geographical locations), data sources (observations, interviews, document reviews), data collectors and analysts. Member-checking was operationalized through the sharing of data interpretations and conclusions with participants for clarifications, corrections and additional information as deemed necessary.

In making sure that the information gathered was consistent and dependable, two researchers (AKA & JKD) conducted a pilot study with the study instrument (checklist) before it was used in the four selected hospitals. Comparison of data collection and thematic analysis was done to ensure consistency of the identified themes. Detailed field notes of the fieldwork also facilitated auditability of the study’s findings. Although qualitative research does not aim at generalization, the settings and procedures involved in the study have been extensively described to enhance transferability of the findings into similar settings.

## Results

### Participant characteristics

The characteristics of the participants have been presented in Table 1.

**Table 1:** Study participants

Group	Observations	Formal Interviews	Informal Interviews	Document audit
Nurses	40	28	40	36
Physicians	12	12	12	36
Hospitalized children	72	20	72	108
Family caregivers	72	20	72	

### Themes

Through an iterative review of the coded field data, the researchers uncovered four prominent and recurring themes which described the culture and context of pediatric pain management in the four included Ghanaian hospitals. These mutually reinforcing themes included the following: “children’s pain expression and response of caregivers”, “pharmacological pain management practices and attitudes”, “managing pain without drugs” and “communication and interaction between pain actors”.

### Children’s pain expression and response of caregivers

One of the recurring themes of the current study was “children’s pain expression and response of caregivers”. Pain was a common complaint among hospitalized children who could verbally communicate. Different levels of pain (mild, moderate and severe) were exhibited by the children at the time of their admission and intermittently during hospitalization. The pain experienced was caused by their medical conditions (such as fractures, sickle cell disease) and or skin breaking procedures (such as intramuscular injections, intravenous cannulation and medication, lumbar puncture, surgical operation, wound dressing among others). For neonates, non-verbal and unconscious children, their parents were of the opinion that the children were in pain during such procedures and wished analgesia was administered. A father of a three-weeks old girl was heard complaining: “Is the pricking not enough, why should a newborn suffer like this”. Children’s incomplete *bodily capital* thus served as a source of discomfort and subsequently subjected them to such discomforting procedures.

As a result of the pain experienced during skin breaking procedures, active and verbal children cried loudly and clung unto their family caregivers for relief. In such cases, caregivers served as a form of *social capital* for the children in distressing times. Nevertheless, there was no escape from pain as healthcare providers regarded the procedures to be of important value and went ahead with them even if it meant applying physical restraints to these children. Some neonates were also observed to squirm and cry during skin breaking procedures. The high level of respect (*symbolic capital) given* to these procedures influenced healthcare providers’ *habitus* of restraining these helpless children. Some family caregivers felt helpless and even cried as they witnessed their children go through the pain associated with skin breaking procedures and some clinical conditions. In some cases, family members could not withstand the traumatic experience their children were going through and excused themselves during such procedures, leaving the children solely in the care of the healthcare providers. A mother of a 6-month old baby expressed it this way “Madam I cannot watch, it makes me sad to watch my child in pain like that”. The children were also observed to exhibit the non-cooperative behaviours that were demonstrated during the skin breaking procedures in subsequent non-painful procedures such as checking of their body temperature, auscultation of their heart and breath sounds among others. According to the children, they kept memories of these painful events which sometimes prevented them from co-operating with healthcare providers during subsequent procedures. The recollection of their previous personal experiences thus influences their noncooperative *habitus*.

Healthcare providers admitted to difficulties in assessing pain among children who could not verbally communicate as a result of their developmental stage or medical condition. According to the healthcare providers, this could be attributed to their lack of *cultural capital* in this area of pain care. They believed crying was the language of infants and toddlers and did not regard this as a cause for concern among these vulnerable population. Their *habitus* of not doing much in such situations on the *field* resulted from the low *symbolic capital* attributed to children’s cry. They further intimated their lack of access to *cultural capital* as they experienced difficulties in distinguishing crying due to pain from other causes. On the other hand, some healthcare providers believed children, especially neonates were more likely to be in pain if the assessment findings of the four traditional vital signs (temperature, pulse, respiration and blood pressure) were higher than the normal range of values. Healthcare providers heavily relied on family caregivers as a source of *social capital* in determining pain and its causes among the children. Whilst some family caregivers reported of using changes in their children’s activity, behaviour and verbalizations to detect pain, others admitted to being clueless in obtaining a definite sign or symptom of their children’s pain. A father of an 18-month child had this to say, “Hmmm Madam he’s been moaning and turning aaarrrhhh... but I can’t tell if he’s feeling any pain though he cries intermittently too”. Hence, *social capital* had a dualistic role in the *field*: serving as a catalyst for pediatric pain assessment on one hand or not favourable on the other hand. Family caregivers and healthcare providers believed children’s expression of the extent of their pain was dependent on the child’s age which served as a source of *cultural capital* and their lack of access to *bodily capital* due to their medical conditions. A nurse puts it in this way:

*“Oh the pain, it usually depends on the condition of the child, what the child will bring in … so maybe if a child has trauma, maybe a child is involved in an accident, compared to a child who has a mild fever or malaria, the pain would be different. So it depends on the condition that, the child would bring.”*

The mother of an a10 month and 6 year-old boys who were both on admission said:

*“As for the pain it depends. The older one will scream when in pain. But look at this little one. He is just whimpering. Maybe it is because he doesn’t understand what is happening to him, compared to the older one”*

Healthcare providers’ individual *habitus* of not prioritizing pain was influenced by the collective *habitus* operating in the field as pain was not actively assessed compared with the other four traditional vital signs (temperature, pulse, respiration rate and blood pressure). Additionally, there were no specially designed documentation sheets for pain, nor was pain assessment pages or sections integrated into the patient’s folders, unlike the four traditional vital signs. This could be attributed to the *symbolic capital* given to this aspect of pain care and the institution’s lack of access to *economic capital* in providing these resources. Thus, pain documentations if any only occurred in the physicians’ and nurses’ notes. An example of such report was:

*“Patient complained of severe headache, 375mg of intravenous Paracetamol administered. Vital signs checked and recorded. Patient tepid sponged to reduce the recorded temperature of 39.6°C to 37.8°C after 40 minutes. Child is made comfortable in bed and mother reassured”*

Observational data from the field work also revealed the presence of two pain assessment tools (Faces Pain Scale and FLACC-Faces, Leg, Activity, Crying, Consolability Scale) in only one of the four hospitals. The few pain assessment tools were also not used by the healthcare providers in assessing children’s pain. The hospitals’ lack of access to *economic capital* in purchasing these assessment tools influenced healthcare providers’ *habitus* of not systematically assessing children’s pain and its subsequent lack of communication during ward rounds and handing over activities. Some healthcare providers were not familiar with pain assessment tools due to their own deficiency of *cultural capital* and the institutions’ lack of *economic capital*; both of which underpinned their *habitus* of assessing children’s pain in a suboptimal manner on the *field*. Some of the healthcare providers also attributed their *habitus* of not using standardized tools in guiding their pain assessment of children to heavy workload and shortage of staff in the demanding pediatric care *field* due to deficiencies in both *cultural capital* and the institutions’deficiency in accessing *economic capital*.

### Pharmacological pain management practices and attitudes

Another recurring theme from the coded field data was “pharmacological pain management practices and attitudes”. Pharmacological interventions served as the mainstay *habitus* in the pain management of children of all ages due to high *symbolic capital* given to such interventions. The common pharmacological agents used in managing children with mild to moderate pain were Paracetamol, Diclofenac and Ibuprofen (Non-Steroidal Anti-Inflammatory Drugs – NSAIDs) which were administered via enteral, parenteral and anal routes. These drugs were alternated in some circumstances due to their mechanism of action and to reduce their side effects. According to the healthcare providers, children who were admitted on account of burns were given Ibuprofen syrup or Paracetamol suppository 30 min before wound dressing to reduce pain. This *habitus* of healthcare providers on the *field* was influenced by their desire to reduce children’s pain and discomfort during such procedures.

Physicians’ *habitus* of seldomly prescribing opioids for children in pain even when it was the best medication in a particular situation was influenced by different forms of *capital* operating on the *field*. Some of these include: the *symbolic capital* associated with these class of analgesics, limited access to *cultural capital* on opioids, influence of other healthcare providers who served as a source of *social capital*, the institutions’ lack of *economic capital* in providing these medications and some families inability to afford these relatively expensive pain medications due to their deficiency in *economic capital*. Nurses’ *habitus* of infrequent administration of prescribed opioids for children were also underpinned by the above-mentioned forms of capital on the pediatric care *field*. Thus, opioid medications (Morphine, Pethidine and Tramadol) which were to be administered when necessary ended up not being given at all even if the child’s condition demanded it. According to the nurses, they deliberately gave other analgesics even when physicians had prescribed opioids. Some nurses further confessed that they have never administered an opioid in their practice even when it was prescribed.

“*I know we are to give Pethidine or morphine, but I have never administered some for any child. After surgery, they come with Pethidine to be given where necessary, but they even do well with ibuprofen and Paracetamol administered intermittently so most times we don’t give. You know, it comes with its own issues.” (Nurse 2, hospital A)*

The habitus of not prescribing and providing opioids (such as Morphine) to children and their families to be taken at home was underpinned by healthcare providers’ prejudiced *symbolic capital* associated with these analgesics and their insufficient *cultural capital* on these drugs. In spite of misconceptions associated with opioids, many healthcare workers admitted that they have not witnessed children experiencing side effects of opioids in their practice during the interview sessions. A physician expressed it this way “I am not sure I have encountered any side effects from the opioids”. A review through the hospital records (doctor’s notes, nurses’ notes and report books) also confirmed this as there were no documented evidence of pain medication side effects.

### Managing pain without drugs

Another recurring theme from the field data was “managing pain without drugs” which largely interconnects with the previous theme of “pharmacological pain management practices and attitudes”. Some of the pain experienced by the children were managed without drugs on the *field,* especially pre-verbal children. Both healthcare providers and family caregivers served as a source of *social capital* for the children and were observed to be working together to manage the pain of the children in this regard. Healthcare providers and family caregivers were observed to be engaged in the *habitus* of using nonpharmacological methods such as cuddling, stroking, coaxing, consoling, positioning and breastfeeding in distracting children’s attention from pain during skin breaking procedures or upon self-report of pain from the children. A typical example of this was the observation of a mother who was softly singing and talking to her baby to stop crying after a venipuncture. Some healthcare providers were also observed to be consoling, cuddling and stroking the back of babies who were crying during and after skin-breaking procedures. A classic instance is when a nurse explained to a seven-year old after a wound dressing to stop crying as he will be leaving the hospital soon. She stated: *“Oww don’t cry my darling … I will take you home today”.*

The pediatric care *field* of the hospitals were equipped with television sets, toys, play areas, and colourful wall paintings with child-friendly designs (such as rainbow, cartoons, etc); reflecting the institutions’ ability to access *economic capital* in the provision of these resources. According to healthcare providers, these facilities were meant to distract children’s attention from painful circumstances during hospitalization. Healthcare providers also said that the environmental layout of the ward as a non-pharmacological pain management strategy had the greatest impact on newly admitted children as they tend to engage with them in the earlier stages of admission. They however, felt its effect diminished over time as children stayed in the ward for longer periods.

Conversely, the children and family caregivers expressed happiness and contentment with the availability of these therapeutic nonpharmacological facilities which diverted children’s attention during painful procedures and served as a form of pain relief. Some children were observed playing on wooden and plastic animal toys in the play area of the hospitals. Other children were also handed teddy bears and toys by healthcare providers before skin breaking procedures to distract their minds and subsequently manage the pain associated with such procedures. A nine-year old girl on admission in one of the hospitals said:

*“ … as for the TV it is the best thing here … I don’t like playing with the toys because I am a big girl … the children can play with them.”*

Notwithstanding these, we observed shortfalls in these resources based on the number of admitted children on the *field* which posed as a challenge and influenced children’s *habitus* of using these distraction methods in managing pain. The children further appealed for the provision of additional toys on the *field* due to the insufficient numbers and the bad state of some playing materials; indicating the hospital’s deficiency in accessing *economic capital* in meeting this demand.

### Communication and interaction between pain actors

The final recurring theme was “communication and interaction between pain actors”. Observation of the interactions that occurred among the participating physicians, nurses, children and their families revealed a cordial and respectful engagement with each other. Physicians and nurses had separate ward rounds but there were periods where both groups came together and reviewed the hospitalized children and their families. The physicians and nurses engaged the children and their families by addressing them by their names and asking how they were doing including any pain they were experiencing. Family caregivers whose children were not capable of communicating effectively gave reports on their well-being and asked for clarifications on issues which were unclear to them. Most of the time, family caregivers and nurses informed physicians about children’s pain complaints after which pain medications were prescribed for the children, but no child was observed to directly complain to physicians about pain during routine ward rounds. The nurses also informed their colleagues to reaffirm their pain assessment findings prior to informing the physicians. All of these interactions reflect how children’s access to *social capital* can serve as a powerful tool in influencing pediatric pain assessment and management *habitus*.

Informal conversations with some hospitalized children and their family caregivers unearthed that they were satisfied and happy with how the healthcare providers engaged with them concerning their wellbeing including that of pain. Nevertheless, some children and their family caregivers were unhappy about their short duration of communication with healthcare providers and wished to be engaged more than what they received. In addition, the dissatisfied family caregivers felt that healthcare providers did not inform them about the treatment being prescribed and administered for their children. Resultantly, some healthcare providers were perceived as good whilst others were perceived by the children and their family caregivers as unkind. This further supports the dualistic potential of *social capital* in either facilitating or impeding pediatric pain care communication. An interaction with a mother of a six-year old boy who was unhappy about communication with healthcare providers expressed it this way:

*“Hmmmm, since I came around at 4am, no nurse has personally come to me to interact with me … Madam it’s their work and since other patients equally need their care, I don’t want to be seen as disturbing them, but I wish one of them could come and talk with me.”*

On the other hand, healthcare providers (physicians and nurses) were generally satisfied with the relationship that existed between them and the hospitalized children as well as their families.

The children clung unto their family caregivers in the hospital environment and perceived them as advocates, who made decisions in their best interest. In situations where children were asked questions by the healthcare providers, they turned toward their family caregivers even before giving responses. Thus, family caregivers mainly served as a source of *social capital* and the mouthpiece in identifying pain and its causes among children who could not talk. On the other hand, some family caregivers felt reluctant in constantly reporting their children’s pain for fear of being tagged as “troublesome” or “medical attention seekers”. They therefore preferred to wait for routine ward rounds before relaying any changes in their children’s condition. Again, this reaffirms the dualistic role of *social capital* in working for or against improved pediatric pain care. Family caregivers also assisted with the activities of daily living of the children including bathing, grooming, feeding and playing. They were constantly beside the children and offered support in care giving whenever it was required; reflecting the power of *social capital* on the *field*.

## Discussion

The multi-faceted complexities and dynamic environments within which healthcare systems operate imply that the same interventions are not likely to work in the same manner in different settings [46, 47]. Thus, understanding the culture and context of pain care is critical for the successful development and implementation of a sustainable short-term educational program targeted for nurses on pediatric pain management. The current study, guided by Bourdieu’s theory of practice [39] was grounded in the assumption that the assessment and management of children’s pain is largely influenced by different forms of *capital* and *habitus* operating in a particular *field*. Our findings revealed that the *habitus* of the pediatric pain actors toward pain assessment and management practices were influenced by various forms of *capital (social, cultural, symbolic, bodily and economic)* operating at different levels on the pediatric care *field*.

Children’s incomplete health status (*bodily capital)* caused them pain, resulting from discomforting skin breaking procedures and this stimulated the *habitus* of both children and caregivers to act in particular ways. In this study, pain was a common complaint among hospitalized children as reported in previous studies [1, 48]. Consistent with earlier studies [49, 50], procedural pain was described by the participating children as the most distressing part of the hospitalization process and memories of such events influenced their *habitus* by discouraging them from co-operating in subsequent procedures. This situation also resulted in a distressing and discomforting *habitus* among some family caregivers as reported in a previous study [51].

Although pain is regarded as of high *symbolic capital* and considered as the fifth vital sign by international bodies [52, 53], we found that pain did not receive the same level of attention for assessment as the traditional four vital signs (temperature, respiration, pulse and blood pressure) in these Ghanaian hospitals. This suboptimal attention to pediatric pain was also identified among Norwegian nurses [54], indicating that the inadequate assessment of children’s pain was not limited to this setting. Healthcare providers admitted to not being able to assess pain in non-verbal children due to their lack of *cultural capital* in this regard and heavily relied on the *social capital* provided by family caregivers who were sometimes clueless about how to assess pain in their children. This also reflects the dualistic potential of social capital in either stimulating or impeding optimal pediatric pain care [55].

In spite of the exponential growth in pain assessment tools for diverse categories of children [56, 58], and contrary to findings by Laures et al. [57] in a pediatric unit, few pain assessment tools were available at the pediatric care *field*, and caregivers did not use any verbal scale or prepared scales on sheets of paper. This reflects the institution’s lack of *economic capital* as well as limited *cultural capital of caregivers* in pain assessment. The few tools were also not utilized in practice to assess children’s pain owing to healthcare providers’ deficiencies in *cultural capital*, adding to the numerous published reports of infrequent pain assessments among hospitalized children [59, 60]. Additionally, there existed limited documentation of pain assessments and evaluations in the studied hospitals, which further underscored the low *symbolic capital* given to pain in the Ghanaian setting. While these pain assessment inadequacies are not unique to the included hospitals [61, 62], they are unacceptable and influences the prolonged unnecessary suffering of vulnerable children. The leadership and management of these hospitals and healthcare in general should take advantage of their *symbolic* and *economic capital* to educate and motivate practitioners to prioritize pediatric pain assessments and provide them with the needed pain assessment tools and documentation charts in practice.

The use of NSAIDs served as the mainstay *habitus* in children’s pain management due to high *symbolic capital* given to such interventions on the *field*. The use of these drugs have been established as a safe treatment option for children with mild to moderate pain [1, 63]. Healthcare providers’ *habitus* of seldomly prescribing and administering opioid analgesics was also attributed to various forms of *capital (symbolic, cultural, social and economic)* controlling the pediatric care *field*. Misconceptions on side effects of opioid analgesics (such as drug dependence, respiratory depression and addiction) have been reported to be prevalent among health care providers in earlier studies [64, 65]. However, these fears are usually unfounded and some of the perceived side effects can be alleviated or totally eliminated by using multi-modal pain treatment approaches [66, 67]. Positive changes in the *habitus* of healthcare providers on opioid use must be actively pursued through regular, short-duration educational interventions. They could be educated on ways of mitigating identified risks and side-effects, minimum doses to achieve effective analgesia, regular assessment for opioid side effects or for indications to discontinue treatment, and also provided with practical and published evidences to correct their misconceptions.

Pain actors’ *habitus* of using diverse nonpharmacological methods to manage pediatric pain were influenced by children’s access to *social capital* and the availability of nondrug resources which reflected the field’s access to *economic capital*. Notwithstanding these, healthcare providers reported of the time-consuming nature of some non-drug interventions which prevented them from using such methods. The shortfalls observed in the provision of playing resources on the field also posed as a challenge and influenced children’s *habitus* of using these distraction methods in managing pain.

Additional nonpharmacological interventions were thus desired by the children despite their contentment with the institution’s *economic capital* in providing such resources on the *field*. The use of wall designs, cartoons, toys and other playing materials in the hospital has been noted as a form of active distraction during painful procedures among children [68, 69]. The reported wide range of effective nondrug pain relief methods such as ball squeezing [70, 71], oral glucose administration [72] and musical mobiles [73] should be explored and encouraged in practice as these methods are cheaper, simple, minimally invasive and can serve as useful adjuncts to analgesics [74].

Communication and interaction between the pain actors depicted how children’s access to *social capital* can serve as a powerful tool in influencing pediatric pain assessment and management *habitus* on the *field* [75]*.* The process of optimal pediatric pain management occurs in a context which is reliant on effective communication among key stakeholders where the pain care needs of the child take center stage. Observation of the relationship and interactions that occurred among the participating physicians, nurses, children and their families revealed a cordial and respectful engagement with each other, unlike Clancy’s report of frustration and expression of anger in their treatment of children’s pain among six healthcare providers in sub-Saharan Africa [62]. This notwithstanding, some family caregivers were dissatisfied with the quality of communication existing in the *field*, and therefore desired for more attention. This is unfortunate, as these family caregivers who serve as a source of *social capital* give voice to the pain of their children, provide information for assessment and support pain management modalities [61, 76]. As such, they should be prepared for their role in the assessment and management of children’s pain so that they can be of help in such circumstances. Healthcare providers should be trained to effectively communicate with these important members in order to improve pediatric pain assessment and management. The healthcare providers in this study also complained of heavy workload in the highly demanding pediatric care *field* which prevented them from pain assessment and management interventions which also impacted their communication with some family caregivers. Heavy workloads impact on the ability of healthcare providers to carry out their responsibilities such as assessments, administering treatments, and communicating with clients and family caregivers about progress of treatment [77]. This calls for an improvement in the healthcare provider-patient ratio; additional auxiliary staff can also be added to the healthcare workforce to assist with the non-technical duties so that the professional staff can concentrate on the technical duties and have more time with children and their families.

Measures which were employed to ensure trustworthiness (credibility, conformability, dependability and transferability) in the current study is considered as one of the study’s strengths. The use of Bourdieu’s theory served as a useful lens for the examination of the sociocultural context of pediatric pain assessment and management at the four Ghanaian hospitals. In spite of the above stated strengths, we reckon that the presence of the ethnographers on the field might have influenced the behaviours of the pain actors even though measures such as not recording the earlier field work were done to safeguard against this. Though, the ethnographers were reflective during the generation of themes, we cannot preclude biases inherent in this process.

## Conclusion

The *habitus* of pediatric pain actors toward pain assessment and management practices are influenced by various forms of *capital (social, cultural, symbolic, bodily and economic)* operating at different levels on the pediatric care *field.* This intricate process is heavily influenced in the hospitalized settings by the culture of “how things are done around here”. The current study has provided useful information on the contextual and cultural factors that influence the assessment and management of children’s pain in hospitalized settings. These include the nature of children’s pain experience and associated responses from care givers, pharmacological and nonpharmacological pain management competencies, and communication among relevant stakeholders (patients, family caregivers and health personnel). Quality improvement programs that seek to enhance this area of practice should use the insights obtained in this study to guide the development, implementation and evaluation stages. Continual professional education programs should focus on training healthcare providers to appropriately assess and manage the pain of diverse children along the developmental milestones. Children and family caregivers should be equally educated on their role in pain assessment and management for improved pediatric pain care delivery. Healthcare facilities should be provided with age- and condition-appropriate pain assessment tools, documentation sheets, and pain management resources to enhance pediatric pain care.

It is not necessarily the establishment of the gap which is interesting, but the exploration of why the gap between beliefs and knowledge about pain management, and the performance of it, actually exists. Effective clinical leadership is therefore required if we are keen on improving pain care outcomes for vulnerable children and their families as it influences resource provision and determines the standards for acceptable and unacceptable behaviour through role modelling and expectation setting. This study has further demonstrated the importance of context in influencing healthcare practices.

## Supplementary Information

**Additional file 1: Appendix I.** Interview guide for healthcare providers. **Appendix II.** Interview guide for children who can verbalize. **Appendix III.** Interview guide for family caregivers

## Data Availability

The dataset supporting the conclusions of this article would be provided upon reasonable request from the corresponding author.

## Abbreviation

NSAIDs: Non-steroidal anti-inflammatory drugs

## References

[1] Birnie KA, Noel M, Parker JA, Chambers CT, Uman LS, Kisely SR (2014). Systematic review and meta-analysis of distraction and hypnosis for needle-related pain and distress in children and adolescents. J Pediatr Psychol.

[2] Friedrichsdorf SJ, Sidman J, Krane EJ (2016). Prevention and treatment of pain in children. Otolaryngol Neck Surg.

[3] Rosenfeld RM, Sury K, Mascarinas C (2015). Office insertion of tympanostomy tubes without anesthesia in young children. Otolaryngol Neck Surg.

[4] Stevens BJ, Abbott LK, Yamada J, Harrison D, Stinson J, Taddio A (2011). Epidemiology and management of painful procedures in children in Canadian hospitals. Can Med Assoc J.

[5] Sinatra R (2010). Causes and consequences of inadequate management of acute pain. Pain Med.

[6] Zarpelon A, Cunha T, Alves-Filho J, Pinto L, Ferreira S, McInnes I (2013). IL-33/ST2 signalling contributes to carrageenin-induced innate inflammation and inflammatory pain: role of cytokines, endothelin-1 and prostaglandin E _2_. Br J Pharmacol.

[7] Galozzi P, Maghini I, Bakdounes L, Ferlito E, Lazzari V, Ermani M (2019). Prevalence of low back pain and its effect on health-related quality of life in 409 scholar adolescents from the Veneto region. Reumatismo..

[8] Abu-Saad Huijer H, Sagherian K, Tamim H (2013). Quality of life and symptom prevalence as reported by children with cancer in Lebanon. Eur J Oncol Nurs.

[9] Windsor RS, Corcoran AL, Lenburg CB, Burnside H, Davitz LJ, Tamura T (2015). Relaxation and guided imagery do not reduce stress, pain and unpleasantness for 11- to 12-year-old girls during vaccinations. Pediatr Nurs.

[10] Nelson LP, Gold JI (2012). Posttraumatic stress disorder in children and their parents following admission to the pediatric intensive care unit. Pediatr Crit Care Med.

[11] Forgeron PA, King S, Stinson JN, McGrath PJ, MacDonald AJ, Chambers CT (2010). Social functioning and peer relationships in children and adolescents with chronic pain: a systematic review. Pain Res Manag.

[12] Mathews L (2011). Pain in children: neglected, unaddressed and mismanaged. Indian J Palliat Care.

[13] Ojeda B, Salazar A, Dueñas M, Torres LM, Micó JA, Failde I (2014). The impact of chronic pain: the perspective of patients, relatives, and caregivers. Fam Syst Health.

[14] Goldberg DS, McGee SJ (2011). Pain as a global public health priority. BMC Public Health.

[15] Cousins MJ, Lynch ME (2011). The declaration Montreal: access to pain management is a fundamental human right. Pain..

[16] Lim Y, Godambe S (2017). Prevention and management of procedural pain in the neonate: an update, American Academy of Pediatrics, 2016. Arch Dis Child - Educ Pract Ed.

[17] Spence K, Henderson-Smart D, New K, Evans C, Whitelaw J, Woolnough R (2010). Evidenced-based clinical practice guideline for management of newborn pain. J Paediatr Child Health.

[18] Witt N, Coynor S, Edwards C, Bradshaw H (2016). A guide to pain assessment and management in the neonate. Curr Emerg Hosp Med Rep.

[19] Taddio A, Appleton M, Bortolussi R, Chambers C, Dubey V, Halperin S (2010). Reducing the pain of childhood vaccination: an evidence-based clinical practice guideline (summary). Can Med Assoc J.

[20] Esmaili BE, Stewart KA, Masalu NA, Schroeder KM (2018). Qualitative analysis of palliative care for pediatric patients with cancer at Bugando medical center: an evaluation of barriers to providing end-of-life care in a resource-limited setting. J Glob Oncol.

[21] Hill M, Stevens D (2011). Volunteers who manage other volunteers and the professionalisation of volunteer management: implications for practice. Volunt Sect Rev.

[22] Finley GA, Kristjánsdóttir O, Forgeron PA (2009). Cultural influences on the assessment of children’s pain. Pain Res Manag.

[23] Stevens B, Riahi S, Cardoso R, Ballantyne M, Yamada J, Beyene J (2011). The influence of context on pain practices in the NICU: perceptions of health care professionals. Qual Health Res.

[24] Atran S, Medin DL, Ross NO (2005). The cultural mind: environmental decision making and cultural modeling within and across populations. Psychol Rev.

[25] Laws RA, Kemp LA, Harris MF, Davies GP, Williams AM, Eames-Brown R (2009). An exploration of how clinician attitudes and beliefs influence the implementation of lifestyle risk factor management in primary healthcare: a grounded theory study. Implement Sci.

[26] Carel H, Kidd IJ (2014). Epistemic injustice in healthcare: a philosophial analysis. Med Health Care Philos.

[27] Nortjé N, Albertyn R (2015). The cultural language of pain: a south African study. S Afr Fam Pract.

[28] Fortier MA, Martin SR, Kain DI, Tan ET (2011). Parental attitudes regarding analgesic use for children: differences in ethnicity and language. J Pediatr Surg.

[29] Twycross A, Finley GA (2014). Nurses’ aims when managing pediatric postoperative pain: is what they say the same as what they do?. J Spec Pediatr Nurs.

[30] Kikuta A, Gardezi F, Dubey V, Taddio A (2011). Practices and perceptions regarding pain and pain management during routine childhood immunizations: findings from a focus-group study with nurses working at Toronto public health, Ontario. Can J Infect Dis Med Microbiol.

[31] Ghana Health Service (2017). The health sector in Ghana: facts and figures.

[32] Aryeetey GC, Westeneng J, Spaan E, Jehu-Appiah C, Agyepong IA, Baltussen R. Can health insurance protect against out-of-pocket and catastrophic expenditures and also support poverty reduction? Evidence from Ghana’s National Health Insurance Scheme. Int J Equity Health. 2016;15:116. 10.1186/s12939-016-0401-1 PMC4957846 27449349

[33] Ahenkan A, Aduo-Adjei K (2017). Predictors of patient satisfaction with quality of healthcare in university hospitals in Ghana. Hosp Pract Res.

[34] Elikplim Pomevor K, Adomah-Afari A (2016). Health providers’ perception of quality of care for neonates in health facilities in a municipality in southern Ghana. Int J Health Care Qual Assur.

[35] Kusi Amponsah A, Kyei EF, Agyemang JB, Boakye H, Kyei-Dompim J, Ahoto CK (2020). Nursing-related barriers to children’s pain management at selected hospitals in Ghana: a descriptive qualitative study. Pain Res Manag.

[36] Kusi Amponsah A, Kyei-Dompim J, Bam V, Kyei EF, Oduro E, Ahoto CK, et al. Exploring the educational needs of nurses on children’s pain management: A descriptive qualitative study. Nurs Open. 2020:nop2.459. 10.1002/nop2.459. 10.1002/nop2.459 PMC7113495 32257272

[37] Beckett K, Henderson EM, Parry S, Stoddart P, Fletcher M (2016). A mixed-method study of pain management practice in a UK children’s hospital: identification of barriers and developing strategies to maintain effective in-patient paediatric pain management. Nurs Open.

[38] Kruk ME, Gage AD, Arsenault C, Jordan K, Leslie HH, Roder-DeWan S (2018). High-quality health systems in the sustainable development goals era: time for a revolution. Lancet Glob Health.

[39] Bourdieu P, Nice R (1977). Outline of a theory of practice.

[40] Pole C, Morrison M (2003). Ethnography for education.

[41] McCambridge J, Witton J, Elbourne DR (2014). Systematic review of the Hawthorne effect: New concepts are needed to study research participation effects. J Clin Epidemiol.

[42] Spradley JP, McCurdy DW (1980). Anthropology, the cultural perspective.

[43] Amaechi EC, Fusch PI (2019). Investigators reflections on the process and experience of a mini-ethnographic case study research in Nigeria. Qual Rep.

[44] Leininger MM, Leininger MM, McFarland MR (2006). Ethnonursing: a research method with enablers to study the theory of culture care. Culture care diversity and universality: a worldwide theory of nursing.

[45] Guba EG, Lincoln YS (1989). Fourth generation evaluation.

[46] Lipsitz LA (2012). Understanding health care as a complex system: the foundation for unintended consequences. JAMA..

[47] Kannampallil TG, Schauer GF, Cohen T, Patel VL (2011). Considering complexity in healthcare systems. J Biomed Inform.

[48] Walther-Larsen S, Pedersen MT, Friis SM, Aagaard GB, Rømsing J, Jeppesen EM (2017). Pain prevalence in hospitalized children: a prospective cross-sectional survey in four Danish university hospitals. Acta Anaesthesiol Scand.

[49] McMurtry CM, Pillai Riddell R, Taddio A, Racine N, Asmundson GJG, Noel M (2015). Far From “Just a Poke”. Clin J Pain.

[50] Noel M, Rabbitts JA, Fales J, Chorney J, Palermo TM (2017). The influence of pain memories on children’s and adolescents’ post-surgical pain experience: a longitudinal dyadic analysis. Health Psychol.

[51] Shave K, Ali S, Scott SD, Hartling L (2018). Procedural pain in children: a qualitative study of caregiver experiences and information needs. BMC Pediatr.

[52] Campbell JN (2016). The fifth vital sign revisited. Pain..

[53] Scher C, Meador L, Van Cleave JH, Reid MC (2018). Moving beyond pain as the fifth vital sign and patient satisfaction scores to improve pain care in the 21st century. Pain Manag Nurs.

[54] Smeland AH, Twycross A, Lundeberg S, Rustøen T (2018). Nurses’ knowledge, attitudes and clinical practice in pediatric postoperative pain management. Pain Manag Nurs.

[55] Fry M, Chenoweth L, MacGregor C, Arendts G (2015). Emergency nurses perceptions of the role of family/carers in caring for cognitively impaired older persons in pain: a descriptive qualitative study. Int J Nurs Stud.

[56] Anand KJS, Stevens BJ, McGrath PJ (2007). Pain in neonates and infants.

[57] Laures E, LaFond C, Hanrahan K, Pierce N, Min H, McCarthy AM (2019). Pain assessment practices in the pediatric intensive care unit. J Pediatr Nurs.

[58] Cohen LL, Lemanek K, Blount RL, Dahlquist LM, Lim CS, Palermo TM (2008). Evidence-based assessment of pediatric pain. J Pediatr Psychol.

[59] Harrison D, Loughnan P, Manias E, Johnston L (2009). Utilization of analgesics, sedatives, and pain scores in infants with a prolonged hospitalization: a prospective descriptive cohort study. Int J Nurs Stud.

[60] Stevens BJ, Harrison D, Rashotte J, Yamada J, Abbott LK, Coburn G (2012). Pain assessment and intensity in hospitalized children in Canada. J Pain.

[61] Matula ST, Polomano RC, Irving SY (2018). The state of the science in paediatric pain management practices in low-middle income countries: an integrative review. Int J Nurs Pract.

[62] Clancy MA (2014). Difficulty, despair and hope – an insight into the world of the health professionals treating paediatric pain in sub-Saharan Africa. J Res Nurs.

[63] Levy DM, Imundo LF (2010). Nonsteroidal anti-inflammatory drugs: a survey of practices and concerns of pediatric medical and surgical specialists and a summary of available safety data. Pediatr Rheumatol.

[64] Chiaretti A, Pierri F, Valentini P, Russo I, Gargiullo L, Riccardi R. Current practice and recent advances in pediatric pain management. Eur Rev Med Pharmacol Sci. 2013;17(1):112-26. 23436673

[65] Forgeron PA, Jongudomkarn D, Evans J, Finley GA, Thienthong S, Siripul P (2009). Children’s pain assessment in northeastern Thailand: perspectives of health professionals. Qual Health Res.

[66] Dongara ARR, Nimbalkar SMM, Phatak AGG, Patel DVV, Nimbalkar ASS (2017). An educational intervention to improve nurses’ understanding of pain in children in Western India. Pain Manag Nurs.

[67] White PF. Multimodal analgesia: its role in preventing postoperative pain. Curr Opin Investig Drugs . 2008;9:76–82. http://www.ncbi.nlm.nih.gov/pubmed/18183534. Accessed 12 Dec 2019. 18183534

[68] Kraemer FW (2010). Treatment of acute pediatric pain. Semin Pediatr Neurol.

[69] Kaheni S, Sadegh Rezai M, Bagheri-Nesami M, Goudarzian AH (2016). The effect of distraction technique on the pain of dressing change among 3–6 year-old Cchildren. Int J Pediatr.

[70] Sahiner NC, Bal MD (2016). The effects of three different distraction methods on pain and anxiety in children. J Child Health Care.

[71] Sadeghi T, Mohammadi N, Shamshiri M, Bagherzadeh R, Hossinkhani N (2013). Effect of distraction on children’s pain during intravenous catheter insertion. J Spec Pediatr Nurs.

[72] Kassab M, Sheehy A, King M, Fowler C, Foureur M (2012). A double-blind randomised controlled trial of 25% oral glucose for pain relief in 2-month old infants undergoing immunisation. Int J Nurs Stud.

[73] Ozdemir FK, Tüfekci FG (2012). The effect of using musical mobiles on reducing pain in infants during vaccination. J Res Med Sci.

[74] Wren A, Ross A, D’Souza G, Almgren C, Feinstein A, Marshall A (2019). Multidisciplinary pain management for pediatric patients with acute and chronic pain: a foundational treatment approach when prescribing opioids. Children..

[75] Sieger M, Fritz E, Them C (2012). In discourse: Bourdieu’s theory of practice and habitus in the context of a communication-oriented nursing interaction model. J Adv Nurs.

[76] Ozcetin M, Suren M, Karaaslan E, Colak E, Kaya Z, Guner O (2011). Effects of parent’s presence on pain tolerance in children during venipuncture: a randomised controlled trial. Hong Kong J Paediatr.

[77] Huggins A, Rahman MM, Claudio D, Torma LM (2014). Balancing nurses’ workload to enhance the quality of care in an outpatient cancer clinic. Int J Collab Enterp.

