# Supplementary material for: Dynamics on the field: a focused study on the culture and context of pediatric pain management at four Ghanaian hospitals
Source: BMC Pediatr. 2020 Nov 20;20:529. doi: 10.1186/s12887-020-02399-w (PMC7678185; doi:10.1186/s12887-020-02399-w)
Supplement: Supplementary file 1 — Additional file 1: Appendix I. Interview guide for healthcare providers. Appendix II. Interview guide for children who can verbalize. Appendix III. Interview guide for family caregivers [file 12887_2020_2399_MOESM1_ESM.docx]

**Appendix I: Interview guide for healthcare providers**

**Preliminary Information**

Unit/Clinic: …………………………………… Age (years): ………………………

Gender: ………………………………. Profession: ……………………………………

Rank: ……………………….. Highest educational level: ……………………………

Duration of working years in the profession: ……………………

Duration of working years in the children’s unit/department: ………………………

**Main Interview Questions**

1. What is your view about the experience of pain among hospitalized children in this unit/department?

*Probes*: How common is pain among children? What types of pain do they complain about? Which medical conditions, injuries or procedures are typically associated with pain complains, behaviours or signals from the children?

1. What methods do you use in identifying pain among different categories of children?

*Probes:* Pain assessment methods for preverbal children or those with non-functional speech and those who can verbally communicate effectively)? Do you face any challenge in the assessment of children’s pain? If yes, tell me more about it.

1. How do you manage pain among different categories of children?

*Probes*: What drugs/ medications are commonly used (prescribed or administered)? What non-drug techniques are frequently used in managing children’s pain? Could you please describe your use of drug or non-drug techniques in the management of children’s pain? Are there any examples of your use of drug or non-drug techniques in practice that you could share?

1. How often do you document on children’s pain assessment and / management and what are the details of your documentation?
2. How do you communicate with other healthcare providers, family caregivers and children themselves regarding their experience of pain?

*Probes*: What communication strategies are used? What in your view makes pain communication effective? Have you encountered difficulties in pain communication among the key stakeholders (children and their families, healthcare providers)? If yes, please tell me more about it and how it was handled?

***Thank you for your time and input.***

**Appendix II: Interview guide for children who can verbalize**

**Preliminary Information**

Unit/Clinic: ………………………………… Age (years): ……………………………

Gender: ……………………… Educational level: ……………………………………

Number of siblings: ……………………… Position of Birth: ……………………

**Main Interview Questions**

1. Tell me briefly about what brought you to the hospital.

*Probes*: Tell me more about the medical condition/ injuries/surgery/ procedures for which you have been admitted in this unit/ department.

1. Please tell me about any pain experiences you have had during the current hospitalization.
2. How do you communicate your pain experience to family caregivers (parents, siblings, guardians, etc.) and healthcare providers (physicians, nurses)?

*Probes*: Do you tell others about it or they have to find out from you or actively ask you about your pain?

1. Following pain communication, how do healthcare providers and family caregivers cater for your pain?

*Probes*: Could you please tell me more about any drug or non-drug techniques that are used in managing your pain? What do the nurses, doctors, family caregiver, etc. doctors do for you when you are experiencing pain? What do you do or engage in to manage your pain?

1. How satisfied are you with the information you receive from the healthcare providers (doctors, nurses, etc.) and family caregivers regarding your pain and pain management?

***Thank you for your time and input.***

**Appendix III: Interview guide for family caregivers**

**Preliminary Information**

Unit/Clinic: ……………………………… Age (years): …………………………………

Gender: …………… Relationship to hospitalized child: ……………………………

Marital status: ……………… Highest educational level: ………………………………

Employment status/ type: ………………………………………………

**Main Interview Questions**

1. How long has your child been admitted in this hospital?
2. How do you tell that your child is in pain?
3. Please tell me about the pain experiences of your child during the current hospitalization.
4. How does it feel to see your child in pain?
5. Do you do anything to help when your child’s complaints about pain?

Probe: Do you use any drug or non-drug techniques in the management of your child’s pain?

1. What role do healthcare professionals play in the identification and management of your child’s play?
2. Tell me about the interactions between you, your child and healthcare providers (doctors, nurses, etc.) regarding your child’s pain experience(s) during hospitalization.
3. How satisfied are you with the information you receive from the healthcare providers (doctors, nurses, etc.) and the pain care of your child?

***Thank you for your time and input.***
